# Sitagliptin attenuates sympathetic innervation *via* modulating reactive oxygen species and interstitial adenosine in infarcted rat hearts

**DOI:** 10.1111/jcmm.12465

**Published:** 2014-11-11

**Authors:** Tsung-Ming Lee, Wei-Ting Chen, Chen-Chia Yang, Shinn-Zong Lin, Nen-Chung Chang

**Affiliations:** a Department of Medicine, Cardiology Section, China Medical University-An Nan HospitalTainan, Taiwan; b Department of Medicine, China Medical UniversityTaichung, Taiwan; c Department of Internal Medicine, School of Medicine, Taipei Medical UniversityTaipei, Taiwan; d Neuropsychiatry Center, China Medical University HospitalTaichung, Taiwan; e Graduate Institute of Immunology, China Medical UniversityTaichung, Taiwan; f Department of Neurosurgery, China Medical University Beigan HospitalYunlin, Taiwan; g Department of Neurosurgery, China Medical University-An Nan HospitalTainan, Taiwan; h Division of Cardiology, Department of Internal Medicine, Taipei Medical University HospitalTaipei, Taiwan

**Keywords:** adenosine, arrhythmia, nerve growth factor, myocardial infarction, reactive oxygen species

## Abstract

We investigated whether sitagliptin, a dipeptidyl peptidase-4 (DPP-4) inhibitor, attenuates arrhythmias through inhibiting nerve growth factor (NGF) expression in post-infarcted normoglycemic rats, focusing on adenosine and reactive oxygen species production. DPP-4 bound adenosine deaminase has been shown to catalyse extracellular adenosine to inosine. DPP-4 inhibitors increased adenosine levels by inhibiting the complex formation. Normoglycemic male Wistar rats were subjected to coronary ligation and then randomized to either saline or sitagliptin in *in vivo* and *ex vivo* studies. Post-infarction was associated with increased oxidative stress, as measured by myocardial superoxide, nitrotyrosine and dihydroethidium fluorescent staining. Measurement of myocardial norepinephrine levels revealed a significant elevation in vehicle-treated infarcted rats compared with sham. Compared with vehicle, infarcted rats treated with sitagliptin significantly increased interstitial adenosine levels and attenuated oxidative stress. Sympathetic hyperinnervation was blunted after administering sitagliptin, as assessed by immunofluorescent analysis and western blotting and real-time quantitative RT-PCR of NGF. Arrhythmic scores in the sitagliptin-treated infarcted rats were significantly lower than those in vehicle. *Ex vivo* studies showed a similar effect of erythro-9-(2-hydroxy-3-nonyl) adenine (an adenosine deaminase inhibitor) to sitagliptin on attenuated levels of superoxide and NGF. Furthermore, the beneficial effects of sitagliptin on superoxide anion production and NGF levels can be reversed by 8-cyclopentyl-1,3-dipropulxanthine (adenosine A_1_ receptor antagonist) and exogenous hypoxanthine. Sitagliptin protects ventricular arrhythmias by attenuating sympathetic innervation *via* adenosine A_1_ receptor and xanthine oxidase-dependent pathways, which converge through the attenuated formation of superoxide in the non-diabetic infarcted rats.

## Introduction

Dipeptidyl peptidase-4 (DPP-4) inhibitors are a new class of oral anti-hyperglycemic agents that prolong the bioavailability of the endogenously secreted incretin hormone glucagon-like peptide-1 (GLP-1) and the glucose-dependent insulinotropic polypeptide 1. DPP-4 inhibitors have been validated and approved as drugs that can lower both fasting and postprandial glucose levels and improve islet β-cell function in patients with diabetes 1. DPP-4 is a high-affinity ligand for membrane-bound adenosine deaminase (ADA), facilitating the scavenging of adenosine from the extracellular environment 2. ADA is an enzyme that transforms, respectively, adenosine and deoxyadenosine to inosine and deoxyinosine, a stage of purine metabolism. In the ensuing reaction hypoxanthine is formed. The oxidation of hypoxanthine to xanthine and the oxidation of xanthine to uric acid are catalysed by xanthine oxidase. Thus, the bimolecular complex of DPP-4 and ADA catalyses the irreversible deamination of adenosine to inosine in the purine catabolic pathway and increases uric acid and superoxide anions as end-products 3. ADA activity is increased in myocardial tissues after myocardial infarction (MI) 4. Although ADA blockers have been reported to decrease radical generation and prevent postischemic heart injury 5, there were controversies regarding the cardioprotection of ADA blockers. Some showed that ADA inhibition failed to decrease infarction size in *in vivo* regional models of myocardial necrosis 6. In contrast, others showed a significantly attenuated infarct size after administering ADA blockers 7. The discrepancy may result from the differences in local interstitial adenosine concentration during ischemia, which can be obtained using the microdialysis technique.

Very recently, we demonstrated that oxidative stress is increased and plays a critical role in ventricular remodelling after MI 8,9. During chronic stage of MI, regional increase in sympathetic innervation was commonly observed at the remote zone 10. Increased sympathetic nerve density has been shown to be responsible for the occurrence of lethal arrhythmias and sudden cardiac death in humans 11. Nerve growth factor (NGF) is a prototypic member of the neurotrophin family, members of which are critical for the differentiation, survival and synaptic activity of the peripheral sympathetic and sensory nervous systems 12. The NGF promoter contains activator protein-1 13, which is subjected to redox regulation through its conserved cysteine residue 14. We have demonstrated that superoxide was responsible for sympathetic innervation in infarcted rats 9. An important source of superoxide anion is the sequential metabolism of adenosine *via* DPP-4 and xanthine oxidase to uric acid 3.

Accumulating data have indicated that DPP-4 inhibitors provide cardioprotection in an insulin-independent manner *via* ancillary pathways 1. Previous studies have shown that the DPP-4 inhibitor sitagliptin can reduce myocardial injury and improve cardiac function in the acute settings of ischemia/reperfusion model 15,16. DPP-4 knockout mice showed a smaller infarct and improved survival after left anterior descending coronary artery ligation than the control mice 17. However, sitagliptin attenuated several, but not all, aspects of adverse remodelling in the post-MI setting 18. For example, Yin *et al*. 19 showed that a DPP-4 inhibitor had no substantial protective effects on cardiac function in a long-term cardiac remodelling model following MI. Thus, the cardiac effect of DPP-4 inhibitors appears to be more complicated than originally thought and requires further research. Because of the affinity of DPP-4 for a variety of substrates, DPP-4 inhibitors have the potential to mediate a wide range of pleiotropic effects, such as antioxidation. Administration of DPP-4 inhibitors has been shown to attenuate increased reactive oxygen species (ROS) 20. The DPP-4 inhibitor vildagliptin has been shown to attenuate ROS production during cardiac ischemia-reperfusion injury in normoglycemic pigs 21. However, it remains unknown whether the DPP-4 inhibitor-induced adenosine inhibited NGF expression through attenuated superoxide levels. Furthermore, the effect of the DPP-4 inhibitor on cardiac electrophysiology during ventricular remodelling has never been investigated. Thus, we assessed (*i*) whether chronic administration of the DPP-4 inhibitor sitagliptin can result in attenuated arrhythmias by inhibiting sympathetic innervation, (*ii*) whether long-term inhibition of superoxide results in attenuated hyperinnervation through attenuated expression of NGF, and (*iii*) whether sitagliptin-induced superoxide attenuation is adenosine- and xanthine oxidase-dependent by the use of adenosine A_1_ receptors (A1R) inhibitor and hypoxanthine supplement in a normoglycaemic rat MI model. In this study, we provide evidence that increasing adenosine levels and decreasing xanthine oxidase substrates synergistically inhibited NGF expression through the attenuation of superoxide after MI.

## Materials and methods

### Animals

The animal experiment was approved and conducted in accordance with local institutional guidelines for the care and use of laboratory animals at the China Medical University and conformed to the *Guide for the Care and Use of Laboratory Animals* published by the US National Institutes of Health (NIH Publication No. 85-23, revised 1996).

#### Experiment 1 (*in vivo*)

Healthy non-diabetic Male Wistar rats (300–350 g) were subjected to ligation of the anterior descending artery as previously described 8, resulting in infarction of the LV-free wall. Rats were randomly assigned into groups of either vehicle (saline) or sitagliptin (5 mg/kg/day, Merck, NJ, USA) administered orally by gastric gavage once a day. The dose of sitagliptin has been shown that blood glucose levels would not be different from those in the vehicle-treated group, thus enabling us to assess direct drug effects independently from blood glucose control 22.

The drug was started 24 hrs after infarction, at a time when they could produce maximum benefits 9. The study duration was designed to be 4 weeks because the majority of the myocardial remodelling process in the rat (70–80%) is complete within 3 weeks 23. Sham operated rats served as controls to exclude the possibility that the drugs themselves directly altered sympathetic innervation. In the sitagliptin-treated group, sitagliptin was withdrawn about 24 hrs before the end of the experiments to eliminate its pharmacological actions.

#### Experiment 2 (*ex vivo*)

To test the magnitude and relative importance of enzyme formation *versus* substrate formation in sitagliptin-related superoxide and NGF levels, we used the ADA inhibitor, the A1R inhibitor and hypoxanthine, respectively, in an *ex vivo* model. Four weeks after induction of MI by coronary ligation, infarcted rat hearts were isolated and subjected to no treatment (vehicle), sitagliptin (5 μM), erythro-9-(2-hydroxy-3-nonyl) adenine (EHNA, 250 μM, ADA inhibitor), sitagliptin + 8-cyclopentyl-1,3-dipropyl-xanthine (DPCPX, 100 nM, A1R antagonist), or sitagliptin + hypoxanthine (1 mM). The doses of sitagliptin, EHNA, DPCPX and hypoxanthine have been shown to be effective in modulating biological activities 5,17,24,25. The heart was perfused with a non-circulating modified Tyrode's solution containing (in mM): glucose 5.5, NaCl 117.0, NaHCO_3_ 23.0, KCl 4.6, NaH_2_PO_4_ 0.8, MgCl_2_ 1.0 and CaCl_2_ 2.0, equilibrated at 37°C and oxygenated with a 95% O_2_ to 5% CO_2_ gas mixture 26. The drugs were infused for 60 min. At the end of the study, all hearts (*n* = 5 each group) were used for performing superoxide measurement and Western analysis for NGF protein at the remote zone (>2 mm outside the infarct).

#### Experiment 3 (*ex vivo*)

To evaluate the importance of peroxynitrite, the by-product of ^•^NO and O_2_^•−^, in sitagliptin-related NGF levels, we performed an *ex vivo* experiment. Four weeks after induction of MI by coronary ligation, infarcted rat hearts were isolated and subjected to no treatment (vehicle), sitagliptin (10 μM), or a combination of sitagliptin and 3-morpholinosydnonimine (37 μM, SIN-1, a peroxynitrite generator). Each heart was perfused with the same protocol as experiment 2. The dose of SIN-1 was used as previously described 27. To preclude non-specific actions to SIN-1, the relatively low concentration of SIN-1 was used. At the end of the study, hearts (*n* = 5 per group) were used for Western blot of NGF at the remote zone.

### Hemodynamics and Infarct size measurements

Hemodynamic parameters were measured in anesthetized rats with ketamine-xylazine (90 mg/kg–9 mg/kg) intraperitoneally at the end of the study. A polyethylene Millar catheter was inserted into the LV and connected to a transducer (Model SPR-407; Millar Instruments, Houston, TX, USA) to measure LV systolic and diastolic pressure as the mean of measurements of five consecutive pressure cycles as previously described 9. The maximal rate of LV pressure rise (+dP/*dt*) and decrease (−dP/*dt*) was measured. After the arterial pressure measurement, the interstitial adenosine measurement and electrophysiological tests were performed. At completion of the electrophysiological tests, the atria and the right ventricle were trimmed off, and the LV was rinsed in cold physiological saline, weighed and immediately frozen in liquid nitrogen after obtaining a coronal section of the LV for infarct size estimation. A section, taken from the equator of the LV, was fixed in 10% formalin and embedded in paraffin for determination of infarct size. Each section was stained with hematoxylin and eosin, and trichrome. The infarct size was determined as previously described 9.

### Cardiac microdialysis

After the arterial pressure measurement, the hearts were excised, the aorta was cannulated, and retrograde perfusion was initiated. Each heart was perfused with modified Tyrode solution equilibrated at 37°C and oxygenated with a 95% O_2_/5% CO_2_ gas mixture. The perfusion medium was maintained at a constant temperature of 37°C. After the perfusion of the isolated hearts was completed, hearts were observed for 10 min. to allow stabilization of contraction and rhythm. Previous studies have shown the usefulness of the microdialysis technique in monitoring of regional myocardial interstitial adenosine levels 28. Microdialysis probes (0.25 mm outer diameter, molecular weight cut-off 5000) were placed in the LV. The dialysis probe was perfused with Ringer solution at a rate of 3.0 μl/min. One sample period was 10 min. We measured dialysate adenosine and uric acid levels from remote regions using high-performance liquid chromatography with electrochemical detection 29. Before analysis, dialysate samples were diluted with 0.01% sodium azide to prevent bacteria degradation.

### *Ex vivo* electrophysiological studies

To avoid the confounding effect of central sympathetic activities on pacing-induced ventricular arrhythmias, we used the Langendorff heart. Because the residual neural integrity at the infarct site is one of the determinants of the response to electrical induction of ventricular arrhythmias 30, only rats with transmural scar were included. Programmed electrical stimulation was performed with electrodes sewn to the epicardial surface of the right ventricular out-flow tract. Pacing pulses were generated from a Bloom stimulator (Fischer Imaging Corporation, Denver, CO, USA). To induce ventricular arrhythmias, pacing was performed at a cycle length of 120 msec. (S_1_) for eight beats, followed by one to three extrastimuli (S_2_, S_3_ and S_4_) at shorter coupling intervals. The end-point of ventricular pacing was the induction of ventricular tachyarrhythmia. Ventricular tachyarrhythmias including ventricular tachycardia and ventricular fibrillation were considered non-sustained when it lasted ≤15 beats and sustained when it lasted >15 beats. An arrhythmia scoring system was modified as previously described 23. When multiple forms of arrhythmias occurred in one heart, the highest score was used. The experimental protocols were typically completed within 10 min.

### Real-time RT-PCR of NGF

Real-time RT-PCR was performed from samples obtained from the remote zone with the TaqMan system (Prism 7700 Sequence Detection System, PE Biosystems) as previously described 9. For NGF, the primers were 5′-GCGTACCCTGACACCAATCT-3′ (sense) and 5′-GGCTCCAGAGACAAGAAACG-3′ (antisense). For cyclophilin, the primers were 5′-ATGGTCAACCCCACCGTGTTCTTCG-3′ (sense) and 5′-CGTGTGAAGTCACCACCCTGACACA-3′ (antisense). Cyclophilin mRNA was chosen as the internal standard because it is expressed at a relatively constant level in virtually all tissues. For quantification, NGF expression was normalized to the expressed housekeeping gene cyclophilin. Reaction conditions were programmed on a computer linked to the detector for 40 cycles of the amplification step.

### Western blot analysis of ADA and NGF

Samples obtained from the remote zone at week 4 after infarction. Rabbit polyclonal antibody to NGF (Chemicon, CA, USA; 1:1000) and ADA (Santa Cruz Biotechnology, Inc., Santa Cruz, CA, USA; 1:1000) was used. Western blotting procedures were described previously 9. Experiments were replicated three times and results expressed as the mean value.

### Immunofluorescent studies of tyrosine hydroxylase, growth-associated factor 43 and neurofilament

To investigate the spatial distribution and quantification of sympathetic nerve fibres, analysis of immunofluorescent staining was performed on LV muscle from the remote zone. Papillary muscles were excluded from the study because a variable sympathetic innervation has been reported 31. Paraffin-embedded tissues were sectioned at a thickness of 5 μm. Tissues were incubated with anti-tyrosine hydroxylase (1:200; Chemicon), anti-growth associated protein 43 (a marker of nerve sprouting, 1:400; Chemicon), and anti-neurofilament antibodies (a marker of sympathetic nerves, 1:1000; Chemicon) in 0.5% BSA in PBS overnight at 37°C. The second antibody was monoclonal goat antimouse IgG conjugated to fluorescein isothiocyanate for tyrosine hydroxylase and rhodamine for growth-associated protein 43 and neurofilament. Isotype-identical directly conjugated antibodies served as a negative control.

The slides were coded so that the investigator was blinded to the identification of the rat sections. The nerve density was measured on the tracings by computerized planimetry (Image Pro Plus, Media Cybernetics, Silver Spring, MD, USA) as described previously 9. The density of nerve fibres was qualitatively estimated from 10 randomly selected fields at a magnification of 400× and expressed as the ratio of labelled nerve fibre area to total area.

### *In situ* detection of superoxide

For evaluating myocardial intracellular superoxide production using *in situ* dihydroethidium (DHE; Invitrogen Molecular Probes, Eugene, OR, USA) fluorescence, optimal cutting temperature media-embedded tissues were sectioned (10 μm) at −20°C. After being fixed, tissues were incubated with DHE in PBS (10 μM) in a dark, humidified container at room temperature for 30 min. Generation of superoxide radicals by tissue was measured in an Olympus fluorescent microscope using an excitation filter of 490 nm, and an emission filter of 580 nm. The density of the images was reported as arbitrary units per millimetre square field.

### Laboratory measurements

We measured the DPP-4 activity and active GLP-1 levels in plasma at the end of the study to confirm that continuous administration of sitagliptin was indeed associated with suppression of plasma DPP-4 activity, and increase in the active GLP-1 levels. EDTA plasma was used to measure active GLP-1 (Millipore Corporation, Billerica, MA, USA) and DPP-4 activity (Quantizume AssaySystem, BIOMOL International, Plymouth Meeting, PA, USA). Insulin was measured by ultrasensitive rat enzyme immunoassay (Mercodia, Uppsala, Sweden).

Although cardiac innervation was detected by immunofluorescent staining of tyrosine hydroxylase, growth-associated factor 43, and neurofilament, it did not imply that the nerves are functional. Thus, to examine the sympathetic nerve function after administering sitagliptin, we measured LV norepinephrine levels from the remote zone. The myocardiums from the remote zone were minced and suspended in a 0.4 N perchloric acid with 5 mmol/l reduced GSH (pH 7.4), homogenized with a polytron homogenizer for 60 sec. in 10 vol. Total norepinephrine was measured using a commercial ELISA kit (Noradrenalin ELISA, IBL Immuno-Biological Laboratories Co., Hamburg, Germany).

Superoxide production by myocardium from the remote zone was measured using lucigenin (5 μM bis-N-methylacridinium nitrate, Sigma-Aldrich, St. Louis, MO, USA) enhanced chemiluminescence as previously described 9. The specific chemiluminescence signal was calculated after subtraction of background activity and expressed as counts per minute per milligram weight (cpm/mg).

To estimate myocardial peroxynitrite formation, we measured free nitrotyrosine (as a marker for peroxynitrite formation) by ELISA (Cayman Chemical, Ann Arbor, MI, USA) in myocardial homogenates.

### Statistical analysis

Results were presented as mean ± SD. Statistical analysis was performed with the SPSS statistical package (SPSS, version 12.0, Chicago, IL, USA). Differences among the groups of rats were tested by an anova. In case of a significant effect, the measurements between the groups were compared with Bonferroni's correction. Electrophysiological data (scoring of programmed electrical stimulation-induced arrhythmias) were compared by a Kruskal–Wallis test followed by a Mann–Whitney test. The significant level was assumed at value of *P* < 0.05.

## Results

### Part 1: *In vivo* study (Experiment 1)

Differences in mortality between the two infarcted groups were not found throughout the study. Sitagliptin had little effect on cardiac gross morphology in the sham-operated rats. Four weeks after infarction, the infarcted area of the LV was very thin and was totally replaced by fully differentiated scar tissue. The weight of the LV inclusive of the septum remained essentially constant for 4 weeks between the infarcted groups (Table1). Compared with vehicle-treated infarcted rats in sitagliptin-treated infarcted rats, the maximal rate of LV +dP/d*t* and −dP/d*t* was significantly increased and lung weight/body weight ratio were significantly lower, consistent with favourable LV remodelling. LV end-systolic pressure and infarct size did not differ between the infarcted groups. In spite of no significant differences in plasma glucose levels between the two infarcted groups, insulin concentrations were significantly increased in infracted rats administered with sitagliptin.

**Table 1:** Cardiac morphology, hemodynamics and plasma glucose, GLP-1, DPP-4, insulin, and tissue NE levels at the end of study

Parameters	Sham		Infarction treated with	
	Vehicle	Sitagliptin	Vehicle	Sitagliptin
No. of rats	10	10	12	12
Body weight, g	397 ± 9	385 ± 11	402 ± 12	405 ± 11
Heart rate, bpm	407 ± 11	404 ± 9	405 ± 11	401 ± 13
LVESP, mmHg	98 ± 4	101 ± 7	96 ± 5	97 ± 5
LVEDP, mmHg	4 ± 3	4 ± 3	19 ± 5*	17 ± 5*
+dp/dt, mmHg/sec.	7891 ± 224	8245 ± 282	2592 ± 305*	3283 ± 235*,†
-dp/dt, mmHg/sec.	6822 ± 228	6974 ± 263	2187 ± 227*	2815 ± 243*,†
Infarct size, %	…	…	41 ± 2	42 ± 2
LVW/BW, mg/g	2.47 ± 0.25	2.46 ± 0.29	3.32 ± 0.29*	3.12 ± 0.39*
RVW/BW, mg/g	0.53 ± 0.11	0.49 ± 0.15	1.21 ± 0.13*	1.18 ± 0.12*
LungW/BW, mg/g	4.12 ± 0.32	4.28 ± 0.47	6.48 ± 0.49*	4.54 ± 0.55†
Glucose, mg/dl	88 ± 5	90 ± 4	92 ± 4	89 ± 7
Insulin, μu/ml	15 ± 11	27 ± 16‡	47 ± 9*	63 ± 13*,†
GLP-1, pmol/l	6.2 ± 0.5	15.8 ± 2.2‡	6.7 ± 0.6	17.2 ± 1.2*,†
DPP-4 activity	1.21 ± 0.11	0.49 ± 0.19‡	1.32 ± 0.16	0.42 ± 0.21*,†
NE, μg/g protein	1.19 ± 0.28	1.26 ± 0.13	2.36 ± 0.31*	1.20 ± 0.36†

Values are mean ± sd.

BW, body weight; LungW, lung weight; LVEDP, left ventricular end-diastolic pressure; LVESP, left ventricular end-systolic pressure; LVW, left ventricular weight; NE, norepinephrine levels from remote myocardium; RVW, right ventricular weight.

* *P* < 0.05 compared with respective sham.

† *P *< 0.05 compared with vehicle-treated infarcted group.

‡ *P *< 0.05 compared with vehicle-treated sham.

#### Plasma GLP-1 and DPP-4 activity, interstitial adenosine and uric acid, and myocardial superoxide, nitrotyrosine and norepinephrine levels

Plasma DPP-4 activity and GLP-1 levels were determined to confirm the successful oral delivery of sitagliptin (Table1). This was associated with a significant increase in active GLP-1 level in the sitagliptin-treated group. Plasma DPP-4 activity was significantly reduced by 68% in the sitagliptin-treated infarcted group compared to the vehicle-treated infarcted group.

**Fig 1:**
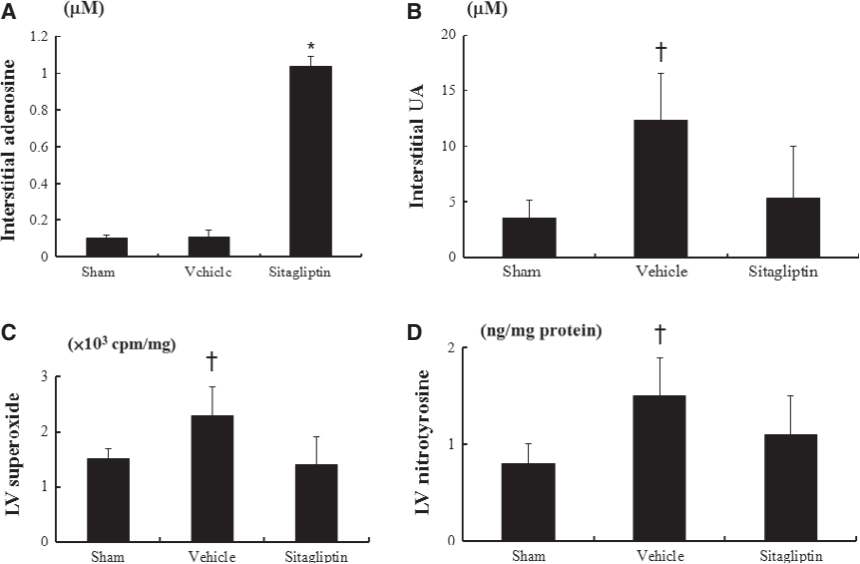
Interstitial (A) adenosine and (B) uric acid and myocardial (C) superoxide, and (D) nitrotyrosine levels from the remote zone. *P < 0.001, compared with sham and the vehicle-treated infarcted rats; †*P* < 0.01, compared with sham and sitagliptin.

Treatment with sitagliptin had a significant increase in interstitial adenosine content compared with vehicle (Fig.1A). Compared with sham, ventricular remodelling after MI was associated with a significant increase in the interstitial concentrations of uric acid, which was attenuated after administering sitagliptin (Fig.1B).

Myocardial superoxide production, as assessed by lucigenin-enhanced chemiluminescence, was markedly increased at the remote zone after MI as compared with sham (*P* < 0.001, Fig.1C). Superoxide was significantly decreased in sitagliptin-treated rats to the level of sham. Myocardial nitrotyrosine in vehicle-treated infarcted rats significantly increased as compared to sham (*P* < 0.001, Fig.1D). Myocardial nitrotyrosine in sitagliptin-treated infarcted rats can be significantly reduced compared with vehicle.

To investigate the possible role of cardiac norepinephrine synthesis, we determined the LV norepinephrine levels. LV norepinephrine levels were significantly up-regulated 1.98-fold in the vehicle-treated infarcted rats in comparison with sham (2.36 ± 0.31 *versus* 1.19 ± 0.28 μg/g protein, *P* < 0.001, Table1). Sitagliptin administration significantly reduced tissue norepinephrine concentrations compared with vehicle-treated infarcted rats.

#### DHE staining in myocardium

**Fig 2:**
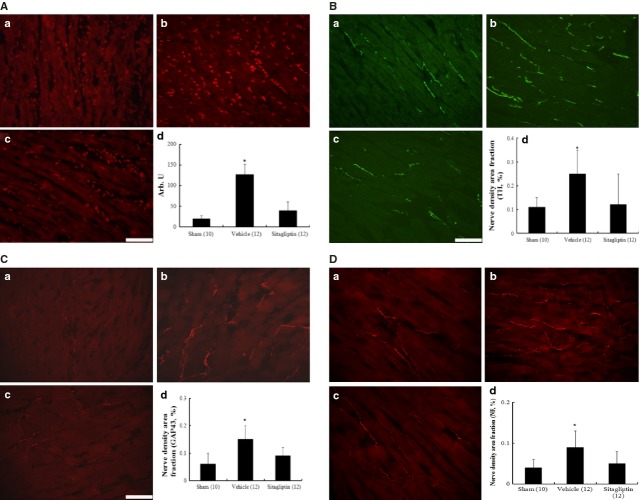
(A) Detection of superoxide in myocardium by DHE staining (magnification 400×). Compared with sham, the DHE fluorescence intensity in the myocardium of the vehicle-treated infarcted group was significantly increased. (B) immunofluorescent staining for tyrosine hydroxylase (TH) from the remote regions. (C) immunofluorescent staining for growth associated protein 43 (GAP43) from the remote regions. (D) immunofluorescent staining for neurofilament (NF) from the remote regions. A, sham; B, infarction treated with vehicle; C, infarction treated with sitagliptin; bar = 50 μm. DHE staining (%) at the remote zone. nerve density area fraction (%) at the remote zone. Each column and bar represents mean ± SD. The number of animals in each group is indicated in parentheses. *P < 0.05, compared with sham and sitagliptin.

DHE reacts with superoxide radicals to form ethidium bromide, which in turn intercalates with DNA to provide nuclear fluorescence as a marker of superoxide radical generation. As shown in Figure2A, post-infarction remodelling markedly enhanced the intensity of the DHE staining at the remote zone in the vehicle-treated rats compared with sham. However, the intensity of the fluorescent signal in the sitagliptin group was significantly reduced relative to the vehicle group.

#### Immunofluorescent analyses

The tyrosine hydroxylase-immunostained nerve fibres appeared to be oriented in the longitudinal axis of adjacent myofibers (Fig.2B). Tyrosine hydroxylase-positive nerve density was significantly increased in the vehicle-treated infarcted rats than that in sham group. Sitagliptin-treated rats show lower nerve density at the remote regions than vehicle-treated rats (0.25 ± 0.10% *versus* 0.12 ± 0.13% in sitagliptin group, *P* < 0.05). Similar to tyrosine hydroxylase results, densities of growth-associated protein 43- (Fig.2C) and neurofilament-positive (Fig.2D) nerves were significantly attenuated in the sitagliptin-treated infarcted rats compared with those in vehicle-treated infarcted group. These morphometric results mirrored those of norepinephrine contents.

#### ADA protein, NGF protein and NGF mRNA expression

**Fig 3:**
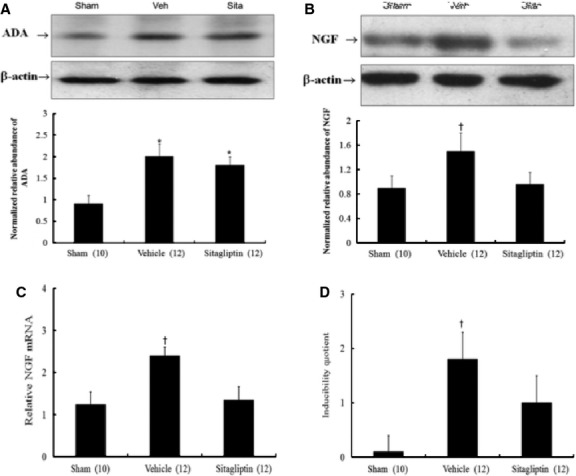
(A) Western blot analysis of ADA (MW: 41 kD) in homogenates of the LV from the remote zone. When compared with vehicle-treated infarcted rats, sitagliptin-treated infarcted rats had significantly lower NGF levels at the remote zone by quantitative analysis. (B) Western blot analysis of NGF (MW: 13 kD) in homogenates of the LV from the remote zone. When compared with vehicle-treated infarcted rats, sitagliptin-treated infarcted rats had significantly lower NGF levels at the remote zone by quantitative analysis. Relative abundance was obtained by normalizing the density of NGF protein against that of β-actin. Results are mean ± SD of 3 independent experiments. (C) Left ventricular NGF mRNA expression. Each mRNA was corrected for an mRNA level of cyclophilin. (D) Inducibility quotient of ventricular arrhythmias by programmed electrical stimulation 4 weeks after MI in an *in vitro* model. Each column and bar represents mean ± SD. *P < 0.05, compared with sham; †*P* < 0.05 compared with sham and sitagliptin.

Western blot shows that ADA and NGF proteins were significantly up-regulated 2.0-fold and 1.5-fold at the remote zone in the vehicle-treated infarcted rats than in sham-operated rats (both *P* < 0.001, Fig.3A and B). When compared with vehicle-treated infarcted rats, sitagliptin-treated infarcted rats had significantly lower NGF levels at the remote zone.

PCR amplification of the cDNA revealed that the *NGF* mRNA levels showed a 1.9-fold up-regulation at the remote zone in the vehicle-treated infarcted rats compared with sham-operated rats (*P* < 0.001, Fig.3C). In sitagliptin-treated infarcted rats, the *NGF* mRNA expression was significantly decreased compared with those in the vehicle-treated infarcted rats.

#### Electrophysiological stimulation

To further elucidate the physiological effect of attenuated sympathetic hyperinnervation, ventricular pacing was performed. Arrhythmia score in sham-operated rats was very low (0.1 ± 0.3; Fig.3D). In contrast, ventricular tachyarrhythmias consisting of ventricular tachycardia and ventricular fibrillation were inducible by programmed stimulation in vehicle-treated infarcted rats. Sitagliptin treatment significantly decreased the inducibility of ventricular tachyarrhythmias compared with those in the vehicle-treated infarcted group.

### Part 2: *Ex vivo* study

#### Effect of A1R and xanthine oxidase signalling on sitagliptin-induced NGF and superoxide levels (Experiment 2)

**Fig 4:**
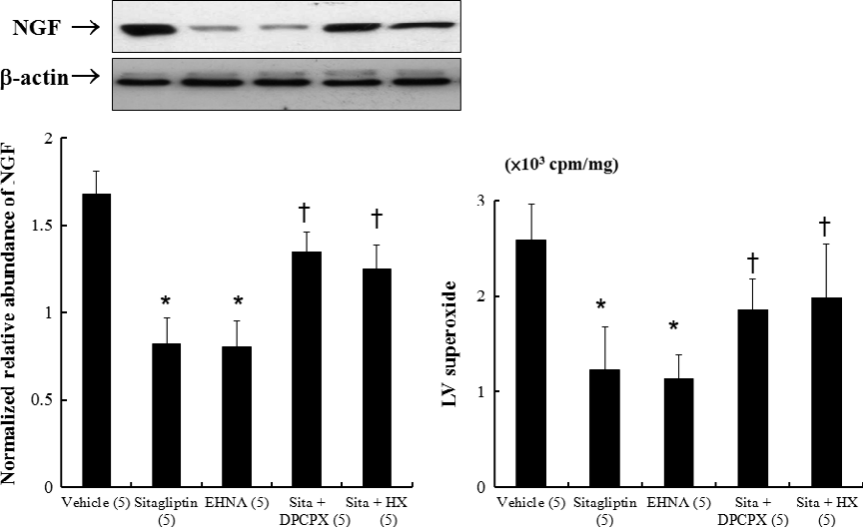
Experiment 2. In a rat isolated heart model, effect of EHNA (ADA inhibitor), DPCPX (A1R antagonist) and hypoxanthine (HX) on NGF and superoxide levels. Compared with sitagliptin-treated infarcted rats alone, significant increased NGF levels and superoxide production was observed in rats treated with DPCPX or hypoxanthine. Relative abundance was obtained by normalizing the density of NGF protein against that of β-actin. Each point is an average of 3 separate experiments (*n* = 5 per group). *P < 0.05 compared with groups treated with vehicle, sitagliptin (sita) + DPCPX, and sitagliptin (sita) + HX; †*P* < 0.05 compared with vehicle.

To test the magnitude and relative importance of enzyme formation *versus* substrate formation in sitagliptin-related NGF and superoxide levels, we perfused the infarcted hearts with EHNA, DPCPX and hypoxanthine. The level of sitagliptin-induced NGF was decreased to 49% over the vehicle (Fig.4), similar to the effect of EHNA. Moreover, this effect of sitagliptin on NGF was significantly inhibited by coadministration of DPCPX (an A1R inhibitor), or hypoxanthine. The changes of superoxide levels were parallel to the NGF levels (Fig.4).

#### Effect of superoxide on NGF levels (Experiment 3)

**Fig 5:**
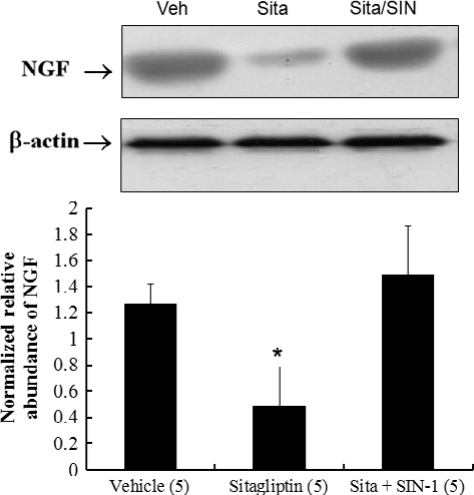
Experiment 3. In a rat isolated heart model, effect of superoxide on NGF levels. SIN-1 significantly increased levels of NGF compared with sitagliptin alone. *P < 0.01 compared with groups treated with vehicle and sitagliptin (sita) + SIN-1.

To elucidate the role of superoxide in modulating NGF, SIN-1 was assessed in an *ex vivo* model. Figure5 shows that SIN-1 significantly increased levels of NGF compared with sitagliptin alone, confirming the role of superoxide in mediating NGF levels.

## Discussions

This study shows for the first time that chronic treatment for 4 weeks with sitagliptin leads to attenuated NGF expression probably through inhibiting superoxide production, independently from its glucose-lowering action. Infarcted hearts during ventricular remodelling deplete the endogenous cardioprotective adenosine levels and leads to the formation of substrates for xanthine oxidase and subsequently enhanced activity of xanthine oxidase occurs which is one of the major sources of ROS. Sitagliptin treatment resulted in two different effects, namely increasing adenosine levels and decreasing xanthine oxidase substrates, in which both attenuated superoxide production. These results were concordant for beneficial effects of sitagliptin, as documented structurally by reduction in cardiac nerve sprouting, molecularly by myocardial NGF protein and mRNA levels, biochemically by interstitial adenosine and uric acid and myocardial superoxide and norepinephrine levels, pharmacologically by DPCPX and hypoxanthine, and electrophysiologically by improvement of fatal ventricular tachyarrhythmias. Adding to the long-standing recognition that A1R activation plays a predominant role in protecting the heart from ischemia-reperfusion, an expanding body of evidence points to the involvement of A1R activation in superoxide production during ventricular remodelling in infarcted rats.

**Fig 6:**
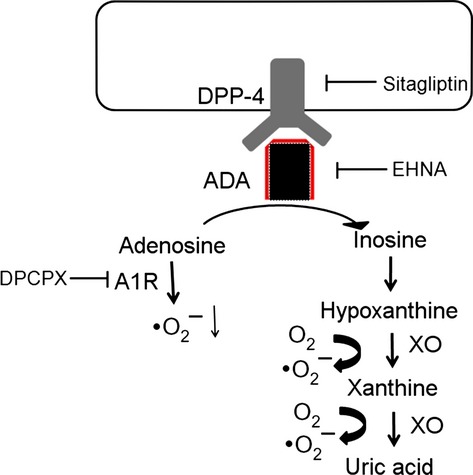
Schematic representation illustrates the superoxide production in post-infarcted rats. Adenosine in the extracellular space is degraded by adenosine deaminase (ADA), which is bound to the cell surface by DPP-4. Adenosine suppresses superoxide production by signalling through adenosine A_1_ receptors (A1R), which can be inhibited by DPCPX. Xanthine oxidase (XO) catalyses the conversion of hypoxanthine, first to xanthine and then to uric acid with superoxide as byproducts.

This study illustrates an additional role of sitagliptin, showing that sitagliptin attenuated sympathetic innervation through coordinated signalling between two distinct pathways, increasing adenosine and decreasing xanthine oxidase substrates, which converge through the attenuated superoxide production. The effect of sitagliptin on attenuated sympathetic innervation was supported by 4 lines of evidence (Fig.6):

1) Substantial evidence indicates that the balance between oxidants and antioxidants is severely disturbed in post-infarcted myocardial tissues 8,9. We reconfirmed that increased ROS levels were observed in infarcted rats as assessed by myocardial superoxide, nitrotyrosine and nuclear oxidative stress as assessed with a DHE staining. Besides, we showed that blocking enzyme by the inhibition of DPP-4 or ADA affected the burst of free radical generation. Finally, this study indicates that dialysate uric acid in hearts with post-MI LV remodelling were significantly increased, which indicates that xanthine oxidase activity may be increased. The results were consistent with the findings of Thompson-Gorman *et al*. 32, showing that that xanthine oxidase is a major source of ROS in rat hearts undergoing ischemia and reperfusion.

2) Sitagliptin administration was associated with increased interstitial adenosine levels. In this study, despite ADA protein levels were significantly increased after MI, no decrease was observed in interstitial adenosine levels. There are several pathways involved in interstitial adenosine formation. Adenosine can be formed *via* dephosphorylation of 5′-AMP by intra- and extracellular 5′-nucleotidases and from *S*-adenosylhomocysteine. Only the ADA bound to DPP-4 on the cell surface was functional and could counteract the inhibitory effect of elevated interstitial adenosine 33. The elevated interstitial adenosine after administering DPP-4 inhibitor was consistent with the observation that the association of DPP-4 with ADA has been shown to lead to activation of the deaminase activity shown by co-immunoprecipitation in rat aortic cells 34. 3) Sitagliptin attenuated the superoxide levels through increasing adenosine and decreasing xanthine oxidase substrates. When DPP-4 is blocked, the net result is a similar degree of reduced ROS generation as that observed with ADA blockade by EHNA. Superoxide levels fell with sitagliptin, an effect abolished by the high-affinity A1R antagonists DPCPX. These data confirm A1R–mediated antioxidation in response to elevated endogenous adenosine. Adenosine receptors represent a family of G-protein coupled receptors that are ubiquitously expressed in a wide variety of tissues. This family contains four receptor subtypes: A_1_ and A_3_, which mediate inhibition of adenylyl cyclase; and A_2A_ and A_2B_, which mediate stimulation of this enzyme. The diverse physiological functions are mediated by the different adenosine receptor subtypes. A1R are primarily localized on cardiomyocytes and contributes to reducing ROS production in hypoxic cardiomyocytes 35. However, conflicting findings were obtained, showing that stimulation of the A_2A_ and A_2B_ adenosine receptor subtypes by exogenous adenosine induces generation of superoxide in the rat cerebral artery 36. Such contrasting actions by adenosine may suggest that this nucleoside could evoke differential regulation of superoxide production in different cell types that might be related to possible differences in the distribution and expression of adenosine receptor subtypes. Indeed, our results were consistent with previous studies, showing that adenosine, acting through its A1R, inhibited adrenergic stimulation and reduced the generation of ROS 37. Although we suggested that sitagliptin provide cardioprotection as an A1R agonist, the improvement of systolic function assessed by peak LV +d*P*/d*t* cannot be explained on the basis of reduced heart rate or reduced systemic blood pressure. Given unlike side effects seen with full A1R agonist, sitagliptin did not induce bradycardia and did not lower systemic pressure. Thus, these differences may favour additional mechanisms unique to sitagliptin that partly drive the observed improvement in LV systolic performance.

To further demonstrate that the beneficial effects of sitagliptin were due to the decreased formation of xanthine oxidase substrates, experiments were performed to determine if the sitagliptin-induced protection could be reversed by hypoxanthine. We observed that an exogenous supply of hypoxanthine abolished the attenuated superoxide production of sitagliptin, implying xanthine oxidase remains active and will still generate considerable ROS in sitagliptin-treated hearts. Thus, it was demonstrated that the burst of radical generation after MI was controlled in part by the formation of enzyme substrates. Given in the presence of its substrate hypoxanthine, xanthine oxidase generates ROS. These results confirm that the sitagliptin-induced protective effects on free radical generation were at least in part because of the decreased formation of xanthine oxidase substrates. The decreased formation of hypoxanthine after administering sitagliptin was further confirmed by our findings, showing a decreased level of interstitial uric acid in infarcted hearts.

4) Sitagliptin attenuated the NGF levels through a superoxide pathway. Sitagliptin is capable of attenuating sympathetic innervation, as indicated with improved fatal arrhythmias. Superoxide might be the mediator responsible for increased NGF levels. SIN-1 significantly increased the attenuated NGF levels in infarcted rat hearts treated with sitagliptin. Peroxynitrite has been shown to activate activator protein-1 activation 38, which in turn to activate the NGF promoter and enhance the transcripts of NGF. These results extended our previous findings that antioxidation by administering N-acetylcysteine or xanthine oxidase inhibitors attenuated sympathetic hyperinnervation after infarction 8,9. The protective effects of sitagliptin were associated with an attenuated myocardial superoxide levels. DPCPX significantly abolished this suppression and augmented NGF levels.

Endogenous adenosine levels increase after administering DPP-4 inhibitors. Adenosine has been shown to exert cardioprotection. Adenosine is either deaminated by ADA or rephosphorylated to 5′-AMP *via* adenosine kinase. Resynthesis of ATP occurs by rephosphorylation of the pool of cytosolic adenosine 39, which cannot be deaminated into inosine in the presence of DPP-4 inhibition. Koshiyama 40 has shown the beneficial effect of the DPP-4 inhibitors with better preservation of cellular ATP levels. Thus DPP-4 inhibition provides cardioprotection by both reducing ROS generation and increasing ATP resynthesis. Furthermore, this elevation of adenosine could have also potentially exerted a protective effect on the heart beyond that which occurs as a result of prevention of ROS generation. It is well known that adenosine is a potent coronary vasodilator, and adenosine-mediated increases in coronary flow could potentially result in enhanced recovery of cardiac function 41. Adenosine can induce vasodilatation by releasing endogenous factors, such as prostacyclin, nitric oxide, endothelium derived hyperpolarizing factor, and epoxyeicosatrienoic acids 42; whether these mediators, by interacting with adenosine, evoke ROS generation in myocardium is not yet known. Taken together, regardless of the relative importance of each of these factors (reduced ROS generation, increased ATP resynthesis, and vasodilatation), all of the DPP-4 inhibitor-caused changes are compatible with our understanding of their protective effects against ventricular arrhythmias.

### Other mechanisms

The mechanisms by which sitagliptin attenuates sympathetic innervation remain to be defined. However, the factor of insulin can be excluded. Insulin secretion significantly increases in rats treated with sitagliptin compared with vehicles as shown in this study. Hyperinsulinemia has been shown to enhance sympathetic innervation 43. The increased insulin levels cannot be a confounding factor of sympathetic innervation because attenuated sympathetic innervation was observed in the group treated with sitagliptin, suggesting that factors other than insulin may contribute to the pathogenesis of sympathetic innervation.

This study suggests that the mechanisms of sitagliptin-attenuated ROS generation are related to increased adenosine levels. However, we cannot rigorously exclude that other potential DPP-4 inhibition might have exerted antioxidant effects on the myocardium such as GLP-1 increase and xanthine oxidase inhibition. First, GLP-1 levels were significantly increased after administering sitagliptin in this study. GLP-1 has been shown to attenuate ROS generation by induction of antioxidant genes 44. Second, linagliptin has recently been shown to attenuate the activity of xanthine oxidase 34. We have demonstrated that the activity of xanthine oxidase positively correlates with ROS generation in infarcted rats 9. Thus, we cannot exclude the possibility that sitagliptin functions similar to linagliptin to attenuated ROS generation *via* direct xanthine oxidase inhibition.

### Clinical implication

DPP-4 inhibitors may possess direct effects on the heart, in addition to potential cardiac benefits possibly mediated through GLP-1. A notable implication of these new findings is that the attenuated adenosine-induced superoxide may regulate myocardial sympathetic innervation. To date, no studies have directly addressed the question of whether or not long-term treatment with sitagliptin may influence the susceptibility to ventricular arrhythmias after MI. In this study, sitagliptin treatment can prevent fatal arrhythmias in non-diabetic rats with normal glucose levels. Given the high risk of coronary artery disease in diabetic patients, the relevance of these potential effects of DPP-4 inhibitors on arrhythmias need to be tested in the clinical setting. Second, after an acute MI, patients remain at high risk for recurrent cardiovascular events and mortality 45. The attenuation of sympathetic hyperinnervation prevents fatal ventricular arrhythmias. Thus, the DPP-4 inhibitors may have important biological effects that prevent the occurrence of post-infarcted arrhythmias. However, very recently, in SAVOR-TIMI 53 trial 46 saxagliptin and in EXAMINE trial 47 alogliptin have shown to be associated with increased incidence of heart failure. Similarly, sitagliptin has shown an association with hospitalization for heart failure in patients recently diagnosed with heart failure 48. Whether this is class effect or just limited to these drugs is not clear. In contrast, in VIVIDD trial vildagliptin showed no increase in incidence of heart failure in patient with LV dysfunction. Furthermore, Takahashi *et al*. 49 have shown that vildagliptin improved cardiac dysfunction in pressure-overloaded mice. It may be that further analysis with DPP-4 inhibitors will give further clarity to the discrepancy.

### Study limitations

There are some limitations in this study that have to be acknowledged. First, the normal daily dose of sitagliptin in the treatment of type 2 diabetes is 100 mg, which corresponds with 1–2 mg/kg per day. In this study, we used a much higher dose (5 mg/kg/day), which did not affect the blood sugar levels. There were differences in median inhibitory concentrations (IC_50_) for DPP-4 and pharmacokinetics among species. For example, sitagliptin inhibited DPP-4 less potently in rats than in humans, with IC_50_ value of approximately 52 nM in rats and 18 nM in humans 50. Furthermore, sitagliptin has a half-life of two hours in rats 51 *versus* 13 hrs in humans 52. Thus, it is not surprising that blood glucose levels were not affected by sitagliptin even at the higher dose (30 mg/kg/day) 53 than that used in this study. The non-glycemic dose was selected so that blood glucose levels would not be different from that in the vehicle-treated group, thus enabling us to assess direct effects of sitagliptin on ROS generation independently from blood glucose control. Second, a non-diabetes animal model was selected for use in this study to discriminate potential pleiotropic effects from those solely attributable to improved blood glucose control. The response of the myocardium to MI may vary with diabetes. Sitagliptin is an antidiabetic agent which exerts its beneficial effects in the cardiovascular system through glycometabolic control. Thus it may be possible that sitagliptin is more beneficial in diabetic models. Further experiments using diabetic animal models are necessary to clearly identify a potentially beneficial effect of DPP-4 inhibition on the attenuation of sympathetic reinnervation.

### Conclusions

These data provide new evidence that sitagliptin protects fatal arrhythmias by attenuating NGF-induced sympathetic innervation *via* inhibiting superoxide production by A1R and xanthine oxidase. The results of the present studies support the concept of pleiotropic antioxidant properties of sitagliptin, suggesting that DPP-4 inhibitors might have antiarrhythmic benefits after MI.
